# Preliminary evidence for vitamin D deficiency in nodulocystic acne

**DOI:** 10.4161/derm.29799

**Published:** 2015-01-14

**Authors:** Mustafa Turgut Yildizgören, Arzu Karatas Togral

**Affiliations:** 1 Department of Physical Medicine and Rehabilitation; Ankara Occupational Diseases Hospital; Ankara, Turkey; 2 Department of Dermatology; Ankara Occupational Diseases Hospital; Ankara, Turkey

**Keywords:** anti-comedogenic effect, comedolytic effect, nodulocystic acne, vitamin D

## Abstract

**Objective:** Acne vulgaris is a chronic inflammatory disease, and hormonal influences, follicular plugging and follicular hyperkeratinization, increased sebum secretion, *Propionibacterium acnes* colonization, and inflammation are involved in its pathogenesis. Recently, a significant body of evidence has accumulated that describes the comedolytic properties of vitamin D and its roles as a modulator of the immune system, a regulator of the proliferation and differentiation of sebocytes and keratinocytes, and as an antioxidant. In this study, we aimed to compare serum vitamin D levels in a group of patients with nodulocystic acne with vitamin D levels in a group of control subjects to determine whether there was any relationship between the vitamin D and acne.

**Methods**: Levels of 25-hydroxyvitamin D (25[OH]D) were measured in 43 patients with newly diagnosed nodulocystic acne and in 46 healthy control subjects, and participants were grouped according to their 25[OH]D levels as follows: normal/sufficient (>20 ng/mL) or insufficient/deficient (<20 ng/mL). Serum concentrations of calcium (Ca), phosphorus (P), alkaline phosphatase (ALP), and parathyroid hormone (PTH) were measured.

**Results:** Forty-three patients and 46 control individuals, with mean ages of 23.13 (± 5.78) years and 25.23 (± 4.73) years, respectively, were included in this study. There were no significant differences between the groups in relation to their body mass indices and Ca, P, ALP, and PTH levels. However, the patients with nodulocystic acne had significantly lower 25[OH]D levels than the subjects in the control group (*P*< 0.05).

**Conclusion**: The patients with nodulocystic acne had relatively low serum vitamin D levels compared with the subjects in the control group. The findings from this study suggest that there is a connection between low vitamin D levels and acne. Larger epidemiologic studies are needed to confirm the status of vitamin D levels in patients with acne.

## Introduction

Acne vulgaris (AV) is a common disorder of the pilosebaceous follicles that affects the skin of the face, neck, upper part of the trunk, and back. Non-inflammatory and inflammatory acne lesions consist of open and/or closed comedones, papules, pustules, and nodules.^1^ The onset of AV usually occurs during adolescence and during early adulthood.^2^ Many factors have been proposed that may underlie the pathogenesis of acne, including hormonal influences, follicular plugs and follicular hyperkeratinization, increased levels of sebum secretion, *Propionibacterium acnes* (*P.acnes*) colonization, and inflammation; recently, AV has also been linked to the insulinotropic or “western diet” and increased mammalian target of rapamycin complex 1 (mTORC1) signaling.^3-5^

Vitamin D regulates the immune system and the proliferation and differentiation of keratinocytes and sebocytes. Furthermore, it has antioxidant and anti-comedogenic properties.^6^ Hence, a vitamin D deficiency may facilitate the pathogenesis of acne. Vitamin D levels have not been investigated in subjects with nodulocystic acne, as far as we are aware. Therefore, the aim of this study was to determine whether there was any relationship between nodulocystic acne and vitamin D levels.

## Results

Forty-three patients (16 men and 27 women) with newly diagnosed nodulocystic acne and 46 skin phototype-matched healthy subjects were included in this study. The mean (± standard deviation [± SD]) age of the patients was 23.13 (± 5.78) years and their mean (± SD) body mass index (BMI) was 22.3 (± 2.6) kg/m^2^. The control group comprised 19 men and 27 women, their mean (± SD) age was 25.23 (± 4.73) years, and their mean (± SD) BMI was 22.8 (± 3.3) kg/m^2^. There were no significant differences between the study groups in relation to age, BMI, and sex distribution. **Table 1** presents the mean (± SD) serum levels of calcium (Ca), phosphorus (P), alkaline phosphatase (ALP), parathyroid hormone (PTH), and 25-hydroxyvitamin D (25[OH]D) for the patient group and the control group. The mean (± SD) concentrations of 25[OH]D in the patient group and the control group were 11.2 (± 5.9) ng/mL and 19.7 (± 8.1) ng/mL, respectively, a difference that was statistically significant (*P* < 0.05). There were no significant differences between the patient and the control groups in relation to any of the other biochemical parameters measured. The prevalence of vitamin D deficiency was 95.3% and 56.5% in the patient group and the control group, respectively, a difference that was statistically significant (*P* < 0.05) (**Table 1**). There were no correlations among the parameters.

**Table 1.** Patient characteristics, the values of biochemical parameters and 25[OH]D classification of both groups

	Case Group (n = 43)	Control Group (n = 46)	*P* value
Sex (Male/Female)	16M, 27 F	19M, 27F	*P* > 0.05
Age (years)	23.1 ± 5.7	25.2 ± 4.7	*P* > 0.05
Height (kg)	64.0 ± 9.2	64.2 ± 12.2	*P* > 0.05
Weight (cm)	169.1 ± 5.8	167.8 ± 7.3	*P* > 0.05
BMI (kg/m^2^)	22.3 ± 2.6	22.8 ± 3.3	*P* > 0.05
Ca (mg/dl)	9.4 ± 0.4	9.4 ± 0.4	*P* > 0.05
P (mg/dl)	3.6 ± 0.5	3.6 ± 0.5	*P* > 0.05
ALP (u/l)	196.3 ± 58	172.9 ± 54	*P* > 0.05
PTH (pg/ml)	36.1 ± 12.0	39.1 ± 13.2	*P* > 0.05
25[OH]D (ng/ml)	11.2 ± 5.9	19.7 ± 8.1	***P*** < **0.05**
25[OH]D (≥ 20/ < 20 ng/ml) < 20 ng/ml (%)	2/41 (95.3%)	20/26 (56.5%)	***P*** < **0.05**

## Discussion

AV is a chronic inflammatory and multifactorial disease.^1^ AV results from androgen-stimulated hyperkeratinization and the obstruction of the pilosebaceous follicles which is secondary to the failure of the normal desquamation of the follicular epithelium, insulin-like growth factor 1 (IGF-1)- and androgen-stimulated excessive sebum production, subsequent colonization of the follicles by *P.acnes*, and, variably inflammation.^7,8^

In our study, the levels of 25[OH]D in the patients with nodulocystic acne were lower than the levels of 25[OH]D in the subjects in the control group. Vitamin D deficiency may be involved in comedogenesis as a consequence of its effects on the proliferation and differentiation of keratinocytes and sebocytes. *P.acnes* growth is exacerbated by the enhanced production of sebum, and this can promote inflammation via toll-like receptor stimulation.^9^ Thus, a vitamin D-deficient state may result in comedogenesis and the exacerbation of inflammation, which characterizes the nodulocystic acne phenotype.

The effect of vitamin D is not only calcium homeostasis, ıt is also important in the regulation of the immune system, cell growth, and cell differentiation.^10^ Human sebocytes and keratinocytes are target cells for biologically active vitamin D metabolites via the nuclear vitamin D receptors (VDRs).^11^ Alterations in the pattern of keratinization within the pilosebaceous follicles result in comedo formation, which is probably the first step in the formation of acne.^1^ It has been reported that 1,25-dihydroxyvitamin D3 (1,25[OH]2D3) suppresses the proliferation and stimulates the differentiation of keratinocytes.^12,13^

Sebocytes and keratinocytes express nuclear VDR and vitamin D-metabolizing enzymes, and both are 1,25[OH]2D3-responsive target cells.^11,14,15^ 1,25[OH]2D3 regulates the growth and differentiation of responsive cells through the nuclear VDR.^16^ Evidence is accumulating that indicates that 1,25[OH]2D3 and forkhead box (FOXO) proteins similarly regulate common target genes. Furthermore, ligand-bound VDR regulates the posttranslational modifications and functions of FOXO proteins.^17^ 1,25[OH]2D3 treatment enhances the binding of FOXO3a and FOXO4 to the promoters of the FOXO target genes, and it interferes with growth factor-induced FOXO protein nuclear export. Moreover, VDR associates directly with the FOXO proteins and their regulators, namely, the sirtuin 1 class III histone deacetylase and protein phosphatase 1. 1,25[OH]2D3 treatment induces FOXO deacetylation and dephosphorylation, which culminates in FOXO activation.^17^ Conversely, FOXO3a phosphorylation is enhanced by the ablation of VDR expression. The 1,25[OH]2D3-induced cell cycle arrest is blocked in FOXO3a-deficient cells, indicating that FOXO proteins are the key downstream mediators of the anti-proliferative actions of 1,25[OH]2D3.^18^

Vitamin D activates FOXO signaling and it also strongly inhibits mTORC1. It has recently been documented that 1,25[OH]2D3 stimulates the messenger ribonucleic acid (mRNA) and protein expression associated with DNA damage-inducible transcript 4 (DDIT4), which is also known as DNA damage response 1.^19^ DDIT4 knockdown by small interfering RNA completely suppressed the anti-proliferative effects of 1,25[OH]2D3.^20^ DDIT4 facilitates the assembly and activation of the tuberous sclerosis complex (TSC)1/TSC2 complex for the eventual suppression of downstream mTORC1 activity.^19^ Hence, mTORC1 appears to be a “master regulator” target for the immunomodulatory and anti-proliferative effects of vitamin D.^21^ One of the most important promoters of comedogenesis is excessive sebum production.^22^ Recent experimental studies indicate that vitamin D analogs may be effective in the treatment of acne.^16,17^

IGF-1 signaling seems to be the major pathway that promotes AV, and it correlates with the clinical course of the disease and the magnitude of sebum production.^23,24^ Indeed, patients with Laron syndrome, which is a congenital IGF-1 deficiency caused by a growth hormone receptor mutation, never present with acne unless they are treated.^25^ IGF-1 enhances peripheral androgen receptor activation via the nuclear extrusion of FOXO1.^8^

It has recently been reported that AV treatments may either increase FOXO1 transcriptional activity or attenuate increased mTORC1 signaling in AV.^18^ The e-supplement accompanying this publication presents evidence to suggest that vitamin D enhances FOXO1 transcriptional activity and downregulates mTORC1, which is the proposed key regulator of cell growth and lipogenesis involved in the pathogenesis of AV, and the authors hypothesize that vitamin D could be a potential new treatment for AV.^18^ 1,25[OH]2D3 suppresses the production of the triglyceride content of sebum via lipogenesis in sebaceous gland cells.^26^

Topically applied active vitamin D3 analogs had comedolytic effects on pseudocomedones in the rhino mouse.^27,28^ Remarkably, serum levels of 1,25[OH]2D3 increased when AV patients were treated with oral isotretinoin.^29^ Hence, the increase in 1,25[OH]2D3 serum vitamin D levels after isotretinoin treatment may suggest another VDR-mediated mechanism that may increase nuclear FOXO levels.

Vitamin D has further inhibitory effects on mTORC1 signaling. At the promoter level, vitamin D induces mitogen-activated protein kinase phosphatase-1 (MKP-1),^30-32^ which is one of the most important factors involved in the inhibition of monocytes and macrophages during the activation of innate immune responses.^33,34^ The vitamin D/MKP-1-mediated attenuation of innate immunity may thus ameliorate tumor necrosis factor (TNF) α signaling, which may lower mTORC1 activity via a reduction in TNF α/IκB kinase β/TSC1 signaling.

Krämer et al.^11^ demonstrated that incubating rapidly proliferating SZ95 sebocytes with 1,25[OH]2D3 resulted in the inhibition of cell proliferation, reductions in the neutral and polar lipid content of cells, and cell cycle regulation by arresting the sebocytes in the G1 phase.^11^ Vitamin D seems to inhibit sebocyte proliferation, differentiation, and sebum secretion, all of which are key factors in the production of sebum.^7^

There is no correlation between serum ALP levels and acne. ALP levels may decrease when vitamin D3 therapy is administered, and this shows similarities with reductions in PTH and Ca levels. In this study, no significant differences were found between the patient and the control groups in relation to the levels of ALP, PTH, Ca, and P.

Taking the results of this study together with the published evidence, vitamin D appears to be intimately involved in the regulation of FOXO1, mTORC1, the innate immune responses, and the inflammation associated with acne, and it warrants greater consideration and further studies to validate these interactions.

## Conclusion

In this study, patients with nodulocystic acne had low vitamin D levels compared with the individuals in the control group. Our study was limited because it was performed during autumn and winter, and these seasons are associated with low levels of vitamin D synthesis. Studies that include larger numbers of participants must be undertaken to demonstrate the relationship between acne and vitamin D more conclusively, and the relationship between vitamin D levels and the severity of acne and/or its characteristics should be evaluated.

## Materials and Methods

This study was undertaken between November 2013 and January 2014. Forty-three individuals who had been diagnosed with nodulocystic acne and 46 skin phototype-matched healthy subjects participated in the study. The local ethics committee approved the study protocol, and it conformed with the ethical guidelines within the 2008 Declaration of Helsinki. The participants’ ages, heights, and weights were recorded, and their BMIs were calculated. Serum concentrations of Ca, P, ALP, PTH, and vitamin D, in the form of 25[OH]D, were measured. The study's exclusion criteria were as follows: receiving therapeutic interventions that might affect vitamin D levels, including bisphosphonates, systemic corticosteroids, vitamin D, and calcium supplements, concomitant inflammatory diseases, inflammatory skin diseases, and the regular use of sunscreen.

Statistical analyses were performed using the IBM® SPSS® software version 20.0 (IBM). Data were expressed as the mean (± SD). The *t* test, chi-square test, and Pearson's correlation analysis were used to statistically analyze the data from the patient and control groups. *P* values of <0.05 were considered statistically significant.
